# Multi-Frequency Asymmetric Absorption–Transmission Metastructures–Photonic Crystals and Their Application as a Refractive Index Sensor

**DOI:** 10.3390/s24196281

**Published:** 2024-09-28

**Authors:** Lei Lei, Xiang Li, Haifeng Zhang

**Affiliations:** College of Electronic and Optical Engineering & College of Flexible Electronics (Future Technology), Nanjing University of Posts and Telecommunications, Nanjing 210023, China; b21021204@njupt.edu.cn (L.L.); b21021012@njupt.edu.cn (X.L.)

**Keywords:** sensor, refractive index detection, metastructure–photonic crystals, asymmetric absorption–transmission

## Abstract

In this paper, a kind of metastructure–photonic crystal (MPC) with multi-frequency asymmetric absorption–transmission properties is proposed. It is composed of various dielectric layers arranged in a periodically tilting pattern. When electromagnetic waves (EMWs) enter from the opposite direction, MPC shows an obvious asymmetry. EMWs are absorbed at 13.71 GHz, 14.37 GHz, and 17.10 GHz in forward incidence, with maximum absorptions of 0.919, 0.917, and 0.956, respectively. In the case of backward incidence, transmission above 0.877 is achieved. Additionally, the MPC is utilized for refractive index (RI) sensing, allowing for wide RI range detection. The refractive index unit is denoted as RIU. The RI detection range is 1.4~3.0, with the corresponding absorption peak variation range being 17.054~17.194 GHz, and a sensitivity of 86 MHz/RIU. By adjusting the number of MPC cycles and tilt angle, the sensing performance and operating frequency band can be tailored to meet various operational requirements. This MPC-based RI sensor is simple to fabricate and has the potential to be used in the development of high-performance and compact sensing devices.

## 1. Introduction

Asymmetric devices refer to electromagnetic waves (EMWs) that exhibit different propagation behaviors when incident from opposite directions, such as absorption, reflection, and transmission [1,2,3]. This bidirectional transmission disparity enables asymmetric devices to find applications in sensing [4], all-optical computing [5], optical signal processing [6], and multiplexing [7]. Researchers have explored various methods to create asymmetry, with building artificial composite structures like asymmetric gratings [8], photonic crystals [9], and metamaterials [10,11] being widely recognized as effective in breaking spatial inversion symmetry [12]. In recent years, the potential use of asymmetric propagation in integrated photonic systems has turned it into a thriving research area. Xu et al. [11] utilized a pair of asymmetric subwavelength gratings and passive hyperbolic metamaterials to achieve visible frequency asymmetry. By coupling the grating in the material’s propagation mode, they achieved asymmetric light transmission with a contrast exceeding 14 dB at central wavelengths of 532 nm and 633 nm. Liu et al. [13] introduced a dual-frequency asymmetric device based on a double-sided composite metal grating, created by connecting a silver grating on the outer surface with an inward inverted T-shaped grating with a Ag film. The device achieved high transmittances of 0.67 and 0.82 at operating wavelengths of 627 nm and 1238 nm, respectively. However, current research primarily focuses on broadband or single-frequency cases, where the opposite direction exhibits similar propagation characteristics. In some other applications, achieving multi-frequency different propagation characteristics of the asymmetric function is crucial.

As a new material design concept, metastructures consist of artificially designed subwavelength units [14] that can acquire properties not found in traditional materials through special multi-scale configuration designs [15], such as a negative refractive index and negative dielectric constant [16]. Due to their unique capability to control the propagation direction, amplitude, and polarization state of EMWs, metastructures can potentially replace traditional bulky optical devices, facilitating the integration and miniaturization of optical devices [17]. Photonic crystals (PCs) can be categorized into one-dimensional, two-dimensional, and three-dimensional structures based on the spatial distribution of dielectric materials [18]. The dielectric constant of PCs exhibits periodicity in space, resulting in a periodic distribution of light refraction. This unique structure gives PCs distinct physical properties like photonic band gaps and photon locality [19], enhancing the interaction between light and matter through effects such as slow optical response [20] and resonator resonance [21]. PCs possess unique capabilities such as reflection, conduction, beam splitting, and coupling to EMWs [22], making them essential materials for micro-nano optoelectronic integration. However, most reported PCs currently suffer from poor structural adjustability and low reflectivity due to their densely packed structure [23]. Metastructures–photonic crystals (MPCs) are a new type of artificial electromagnetic material that combines metastructures with the concept of traditional PCs. By precisely adjusting the geometric and material properties of MPC, its flexibility and energy localization can be enhanced to achieve a specific response to EMWs.

Because of their small size, high sensitivity, and strong resistance to electromagnetic interference, optical sensors have garnered significant attention from researchers, making them a focal point in refractive index (RI) measurements in recent years [24,25,26]. Currently, various physical mechanisms have been utilized to improve sensing performance, such as surface plasmon resonance [27], Fano resonance [28], and PC defect mode resonance [29,30]. In 2016, Chen et al. [27] first proposed a liquid-core optical fiber surface plasmon resonance sensor with an adjustable nanoporous silica coating. By adjusting the RI of the nanoporous silica coating, the sensor can be used for different RI detection ranges. In 2019, Elnaz et al. [26] proposed a tunable polarimetric sensitive RI sensor with a sensitivity of up to 10,000 nm/RIU and a sensitivity that can be adjusted based on the surface conductivity of graphene-based chiral structures. In 2023, Li et al. [31] proposed a sensor based on hyperbolic metamaterials, which offers an adjustable index interval of analytes for detecting biochemical molecules in the terahertz band. In addition, the specialized design of superstructures to enhance absorption or transmission has been extensively studied for their application in refractive index detection. Li et al. [32] utilized a graphene-based silicon carbide grating and Tamm plasmon structure to achieve a dual-band absorption peak that is highly sensitive to the refractive index of the air layer. Hu et al. [33] excited the Tamm plasmon polariton resonance based on a graphene-based hybrid Tamm plasmon structure to develop a multi-channel absorption and refractive index sensor. These studies play a crucial role in enhancing absorption or transmission, providing significant advancements in the field of optical sensing. Due to the rapid development of the biochemical industry, the demand for sensing and detection continues to rise. Therefore, it is necessary to promote the use of sensing materials and diversify device design.

In this paper, a kind of plasma-based MPC is proposed to achieve the asymmetric propagation of EMWs and wide-range RI detection, enriching the design possibilities for asymmetric devices and sensors. The MPC consists of materials with varying RIs arranged periodically at an oblique angle. Due to the asymmetrical spatial distribution of the MPC, EMW propagation characteristics differ significantly depending on the incident direction, exhibiting a noticeable asymmetric absorption–transmission (AAT) phenomenon. When EMWs are incident in the forward direction, a high absorptivity of 0.94 is achieved at the operating frequency of 24.92 GHz, while in the backward case, a high transmittance of 0.98 is achieved. Even after adding an analyte layer, the AAT phenomenon persists and exhibits multi-frequency behavior. At frequencies of 13.71 GHz, 14.37 GHz, and 17.10 GHz, the forward absorptivity values are 0.919, 0.917, and 0.956, respectively, with backward transmittance above 0.877. To showcase the superiority of the proposed MPC, RI sensing is conducted, demonstrating a wide RI range of 1.4 to 3.0. The absorption peak corresponding to the RI values shifts from 17.194 GHz to 17.054 GHz, with a sensitivity of 86 MHz/RIU. Furthermore, this paper discusses in detail the effects of cycle number and tilt angle on MPC performance, considering potential errors in MPC fabrication and practical usage. The proposed MPC offers advantages such as multifunctionality, compact size, large measurement range, and suitability for asymmetric device design and biosensing applications.

## 2. The Theoretical Model

**Figure 1:**
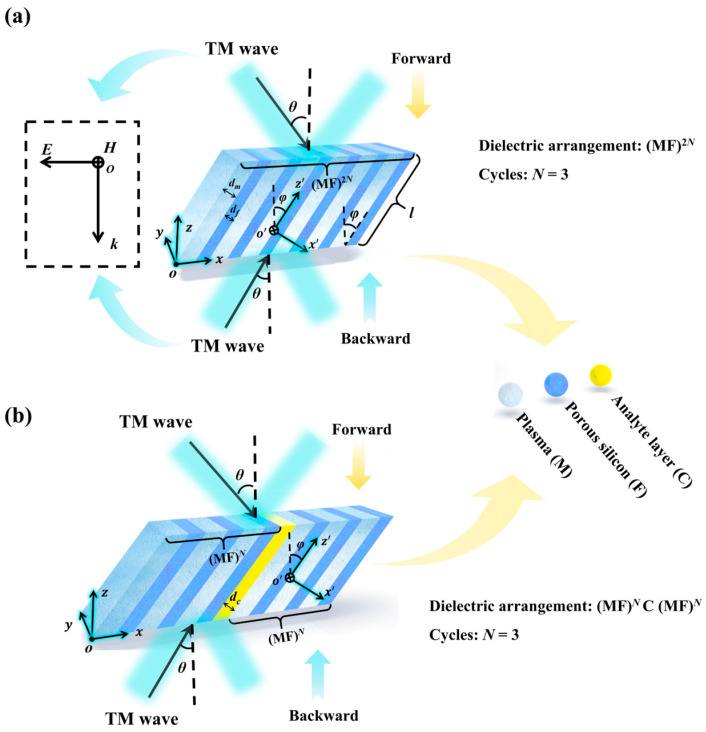
Schematic diagram of the MPC: (**a**) ordinary periodic structure; (**b**) introducing the analyte layer.

The schematic diagram of the proposed MPC is shown in Figure 1. We use M to represent the plasma layer, F to represent the porous silicon layer, and *N* = 3 to represent the number of periods. In Figure 1a, in the ordinary structure, M and F are arranged periodically, and the arrangement rule is (MF)^2*N*^. In Figure 1b, the analyte layer (C) is introduced into the original structure, breaking the periodicity of the structure, and its arrangement rule is (MF)*^N^*C(MF)*^N^*. Two coordinate systems (*x*, *y*, *z*) and (*x’*, *y’*, *z’*) are defined in Figure 1. The coordinate system (*x’*, *y’*, *z’*) is obtained by rotating the coordinate system (*x*, *y*, *z*) clockwise by an angle of *φ* centered on the *y*-axis. Two arrows in different directions at the top and bottom indicate the forward and backward incidence of EMWs. In this paper, the initial condition set by incident EMWs is transverse magnetic (TM) wave [34], and TM mode propagation means that there is no magnetic field component in the propagation direction. The background is air, and the permittivity of layers M, F, and C is expressed by *ε_m_*, *ε_f_*, and *ε_c_*, respectively, where *ε_f_* = 1.3 and *ε_c_* = 3.7 [35]. The thickness is indicated by *d_m_*, *d_f_*, and *d_c_*. In Figure 1a, *d_m_* = 0.4 mm, *d_f_* = 1.0 mm, and the length of the hypotenuse *l* = 20 mm. In Figure 1b, *d_m_* = 0.6 mm, *d_f_* = 1.0 mm, *d_c_* = 1.0 mm, *l* = 20 mm. *φ* is the inclination angle of the MPC with respect to the *z*-axis, *θ* is the incidence angle of the TM wave, and the specific values are *φ* = 40°and *θ* = 50°. Related parameter settings are shown in Table 1.

Due to the introduction of the magnetic field, the plasma is seen as an anisotropic medium, and its dielectric function is described as follows [36]:

(1) $$\varepsilon_{m}=\frac{\varepsilon_{1}^{2}-\varepsilon_{2}^{2}}{\varepsilon_{1}}$$

(2) $$\varepsilon_{1}=1-\frac{\omega_{p}^{2}(\omega +i\nu_{c})}{\omega [{\left(\omega +i\nu_{c}\right)}^{2}-\omega_{c}^{2}]}$$

(3) $$\varepsilon_{2}=-\frac{\omega_{p}^{2}\omega_{c}}{\omega [{\left(\omega +i\nu_{c}\right)}^{2}-\omega_{c}^{2}]}$$

where *ω_p_* = (*e*^2^*n_e_*/*mε*_0_)^1/2^ is the plasma frequency, *ω* is the frequency of the incident TM wave, *ν_c_* is the collision frequency of electrons, *ω_c_* = (*eB*/*m*) is the plasma electron oscillation frequency, *m* is the mass of the electron, *e* is the charge of the electron, and *n_e_* is the plasma density. Their respective values are as follows: *B* = 0.1 T, *n_e_* = 10^18^ m^−3^, and *ν_c_* = 0.1*ω_p_* [36].

In the ordinary periodic structure, depicted in Figure 1a, the effective relative permittivity tensor of the MPC in the coordinate system *x*’*y*’*z*’ is [37]

(4) $$\varepsilon^{\prime }=\left[\begin{array}{ccc} {\varepsilon_{xx}}^{\prime } & 0 & 0\\ 0 & {\varepsilon_{yy}}^{\prime } & 0\\ 0 & 0 & {\varepsilon_{zz}}^{\prime } \end{array}\right]$$

According to the effective medium model [38], MPC can be described as a uniaxial anisotropic medium. *ε_xx_*’ represents the component of the permittivity tensor parallel to the *x*’-axis, while *ε_yy_*’ and *ε_zz_*’ are the components of the permittivity tensor parallel to the *y*’-axis. Refer to the following formula for the specific form [38]:

(5) $$\frac{1}{\varepsilon_{xx}\prime }=\frac{1}{d_{m}+d_{f}}(\frac{d_{m}}{\varepsilon_{m}}+\frac{d_{f}}{\varepsilon_{f}})$$

(6) $$\varepsilon_{yy}\prime =\varepsilon_{zz}\prime =\frac{d_{m}\varepsilon_{m}+d_{f}\varepsilon_{f}}{d_{m}+d_{f}}$$

The relation between the coordinate system *xyz* and the coordinate system *x*′*y*′*z*′ is as follows [37]

(7) $$\left[\begin{array}{c} x\\ y\\ z \end{array}\right]=\left[\begin{array}{ccc} \cos\varphi & 0 & \sin\varphi \\ 0 & 1 & 0\\ -\sin\varphi & 0 & \cos\varphi \end{array}\right]\left[\begin{array}{c} x^{\prime }\\ y^{\prime }\\ z^{\prime } \end{array}\right]$$

The effective relative permittivity tensor of the MPC in the coordinate system *xyz* is

(8) $$\varepsilon =\left[\begin{array}{ccc} \varepsilon_{xx} & 0 & \varepsilon_{xz}\\ 0 & \varepsilon_{yy} & 0\\ \varepsilon_{zx} & 0 & \varepsilon_{zz} \end{array}\right]$$

where *ε_xx_*, *ε_yy_*, and *ε_zz_* can be represented as

(9) $$\begin{array}{l} \varepsilon_{xx}=\varepsilon_{xx}\prime \cos^{2}\varphi +\varepsilon_{yy}\prime \sin^{2}\varphi \\ \varepsilon_{zz}=\varepsilon_{xx}\prime \sin^{2}\varphi +\varepsilon_{yy}\prime \cos^{2}\varphi \\ \varepsilon_{xz}=\varepsilon_{zx}=\left({\varepsilon_{yy}}^{\prime }-{\varepsilon_{xx}}^{\prime }\right)\sin\varphi \cos\varphi \end{array}$$

In the coordinate system *x*′*y*′*z*′, the electric displaces vector **D**′, and electric filed vector **E**’ is

(10) $$D^{\prime }=\varepsilon_{0}\varepsilon^{\prime }E^{\prime }$$

In the coordinate system *xyz*, it is

(11) $$D=\varepsilon_{0}\varepsilon E$$

The magnetic field has only *y*-directional components for both the forward and backward incidence of the TM waves, so [37]

(12) $$H_{y}=H_{y}^{+}\vec{y}+H_{y}^{-}\vec{y}=H_{y_{0}}^{+}e^{i\left(k_{z+}\cdot z+k_{x}\cdot x-\omega t\right)}\vec{y}+H_{y_{0}}^{-}e^{i\left(k_{z-}\cdot z+k_{x}\cdot x-\omega t\right)}\vec{y}$$

where *k_z_*_+_ and *k_z_*_−_ indicate the +*z* and −*z* components of the TM wave vector.

So, we can obtain [37]

(13) $$\left[\begin{array}{c} D_{x}\\ 0\\ D_{z} \end{array}\right]=\varepsilon_{0}\left[\begin{array}{ccc} \varepsilon_{xx} & 0 & \varepsilon_{xz}\\ 0 & \varepsilon_{yy} & 0\\ \varepsilon_{zx} & 0 & \varepsilon_{zz} \end{array}\right]\left[\begin{array}{c} E_{x}\\ 0\\ E_{z} \end{array}\right]$$

According to Maxwell’s equations, the following equation can be derived

(14) $$\begin{array}{l} k_{z+}H_{y}^{+}+k_{z-}H_{y}^{-}=\omega \varepsilon_{0}\left(\varepsilon_{xx}E_{x}+\varepsilon_{xz}E_{z}\right)\\ k_{x}\left(H_{y}^{+}+H_{y}^{-}\right)=-\omega \varepsilon_{0}\left(\varepsilon_{zx}E_{x}+\varepsilon_{zz}E_{z}\right) \end{array}$$

(15) $$E_{x}=\frac{\varepsilon_{zz}k_{z+}+\varepsilon_{xz}k_{x}}{\omega \varepsilon_{0}\left(\varepsilon_{xx}\varepsilon_{zz}-{\varepsilon_{xz}}^{2}\right)}H_{y}^{+}+\frac{\varepsilon_{zz}k_{z-}+\varepsilon_{xz}k_{x}}{\omega \varepsilon_{0}\left(\varepsilon_{xx}\varepsilon_{zz}-{\varepsilon_{xz}}^{2}\right)}H_{y}^{-}$$

From $\nabla \times E=-\frac{\partial B}{\partial t}$, for the forward incident wave, we have [37]

(16) $$\left(\frac{\partial E_{x}}{\partial z}-\frac{\partial E_{z}}{\partial x}\right)=i\omega \mu_{0}\mu H_{y}^{+}$$

The following result can be obtained by combining the above equation:

(17) $$\varepsilon_{zz}{k_{z}}^{2}+\left(\varepsilon_{xz}k_{x}+k_{x}\varepsilon_{zx}\right)k_{z}+\varepsilon_{xx}{k_{x}}^{2}={k_{0}}^{2}\left(\varepsilon_{xx}\varepsilon_{zz}-{\varepsilon_{xz}}^{2}\right)$$

The two results of solving Equation (17) are as follows

(18) $$k_{z1}=(-\varepsilon_{xz}k_{x}+\sqrt{\left({\varepsilon_{xz}}^{2}-\varepsilon_{zz}\varepsilon_{xx}\right)\left({k_{x}}^{2}-{k_{0}}^{2}\varepsilon_{zz}\right)})/\varepsilon_{zz}$$

(19) $$k_{z2}=(-\varepsilon_{xz}k_{x}-\sqrt{\left({\varepsilon_{xz}}^{2}-\varepsilon_{zz}\varepsilon_{xx}\right)\left({k_{x}}^{2}-{k_{0}}^{2}\varepsilon_{zz}\right)})/\varepsilon_{zz}$$

where *k*_0_ = *ω*/*c* and *k_x_* = *k*_0_ sin*θ* [37].

Equations (18) and (19) reveal the root cause of AAT characteristics. They represent the forward incidence and backward incidence of EMWs, respectively. For ordinary dielectric materials, *k_z_*_1_ and *k_z_*_2_ are inversely related, and the structure can be viewed as reversible. However, in the MPC, *k_x_* is a non-zero constant because the spatial symmetry is broken. Therefore, the values of *k_z_*_1_ and *k_z_*_2_ are no longer negative, resulting in a significant difference. This leads to different propagation modes of EMWs in the forward and reverse incidents [37].

In addition, the quantitative evaluation of RI sensors involves various indicators, such as sensitivity (S), figure of merit (FOM), and quality factor (Q) [39].

One of the metrics used to evaluate the performance of a proposed sensor is FOM. This measurement is calculated by the ratio of the half-peak width to the peak resonance wavelength [39].

(20) $$FOM=\frac{S}{FWHM}$$

Among it, FWHM stands for full width at half maximum, which represents the bandwidth of the RI sensor response and indicates the range of signals that the sensor can effectively detect. A narrower FWHM generally indicates higher sensitivity and a higher FOM.

Q is used to measure the energy stored in the MPC relative to the energy lost through dissipative mechanisms such as absorption and scattering. A high Q value indicates that the MPC performs well in efficiently detecting RI changes in the surrounding environment. Mathematically, Q can be defined as follows [39]:

(21) $$Q=\frac{f_{0}}{FWHM}$$

where *f*_0_ is the resonance frequency.

## 3. Analysis and Discussion

The simulation results presented in this paper were obtained using the HFSS method and calculated through the finite element method. In the simulation, the x- and y-directions are subject to periodic boundary conditions, while the z-direction is treated as an open space.

**Figure 2:**
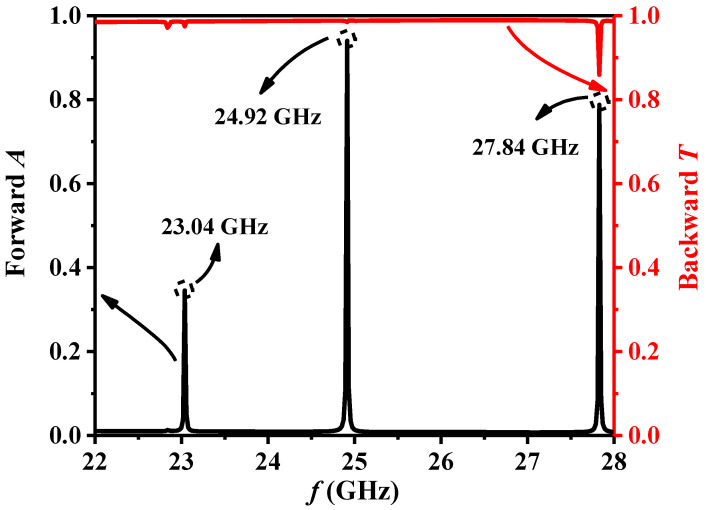
Forward absorption and backward transmission curves in ordinary periodic MPC.

Figure 2 illustrates the EMW propagation characteristic curves corresponding to the ordinary periodic MPC in Figure 1a at frequencies ranging from 22 GHz to 28 GHz. It is evident from the figure that there is a significant difference in the propagation of the MPC when EMWs are incident in the forward and backward directions. Upon forward incidence, EMWs exhibit absorption peaks at 23.04 GHz, 24.92 GHz, and 27.84 GHz, respectively. At the three distinct resonance frequencies, the forward absorption rate can reach 0.34, 0.79, and 0.94, respectively. When the incident light is in the backward case, as Equations (18) and (19), the propagation mode will change. During this scenario, EMWs are predominantly transmitted, with a transmittance exceeding 0.9 within 22~28 GHz. Notably, at *f* = 24.92 GHz, the backward transmittance is 0.98, showcasing a very high forward absorption and backward transmittance, demonstrating excellent AAT characteristics.

**Figure 3:**
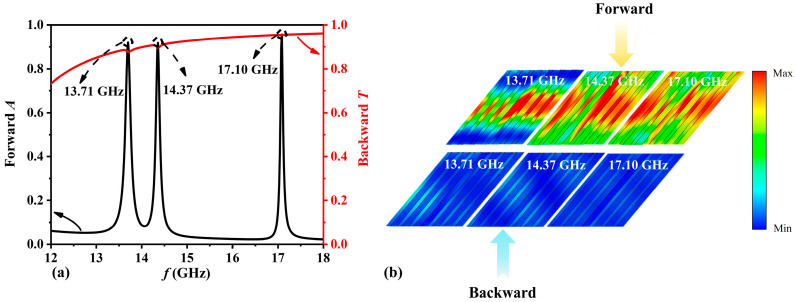
(**a**) Forward absorption and backward transmission curves of the MPC introduced into the analyte layer. The black solid line represents the forward absorption curve, the red solid line indicates the backward transmission curve, and the black dashed arrow marks the resonance frequency. (**b**) Energy distribution of forward and backward incident electric fields at resonance frequency points.

After introducing the analyte layer into the MPC, the structure diagram is depicted in Figure 1b. Figure 3 illustrates the forward absorption and backward transmission curves in this scenario, revealing the continued presence of the AAT phenomenon, which is significantly enhanced. Under forward incidence, EMWs still trigger three absorption peaks at 13.71 GHz, 14.37 GHz, and 17.10 GHz, respectively. However, unlike Figure 2, the absorption rates corresponding to the three resonant frequencies are all above 0.9, specifically 0.919, 0.917, and 0.956, respectively. The backward transmittance rates are 0.877, 0.896, and 0.953, respectively, maintaining high levels. The root cause of the asymmetric propagation illustrated in Figure 3a is explained by Equations (18) and (19). Due to the non-uniform distribution of the MPC dielectric function, the components of the dielectric constant tensor vary in different directions. This variation alters the values of *k*_z1_ and *k*_z2_, causing EMWs to experience different propagation modes when incident from opposite directions. Additionally, the MPC possesses a specific structural eigenfrequency. When the frequency of the incident EMW aligns with this intrinsic frequency, the absorption and transmission of the EMW by the MPC change significantly. For instance, at the resonance frequency, the electric field energy reaches its maximum, making the EMW more readily absorbed and resulting in a peak in the absorption curve. Figure 3b clearly illustrates the energy distribution of EMWs within the MPC, revealing that the area of the concentrated electric field energy corresponds to the trough in the absorption curve. These outcomes demonstrate that the proposed MPC exhibits multi-frequency AAT characteristics following the introduction of the defect layer, offering a fresh approach for designing asymmetric devices across various frequencies.

Currently, RI sensors have increasingly extensive applications in biomedicine, environmental monitoring, and other fields. By detecting changes in the RI of the medium being measured, the resonance condition changes, leading to variations in wavelength or frequency, enabling the detection of RI. The forward absorption depicted in Figure 3 exhibits sharp spikes. Upon calculation, the Q values of the absorption peaks at the resonance frequencies of 13.71 GHz, 14.37 GHz, and 17.10 GHz are 93.3, 131.8, and 229.8, respectively. Notably, the absorption peak at 17.10 GHz boasts the highest Q value, indicating potential for sensor applications.

**Figure 4:**
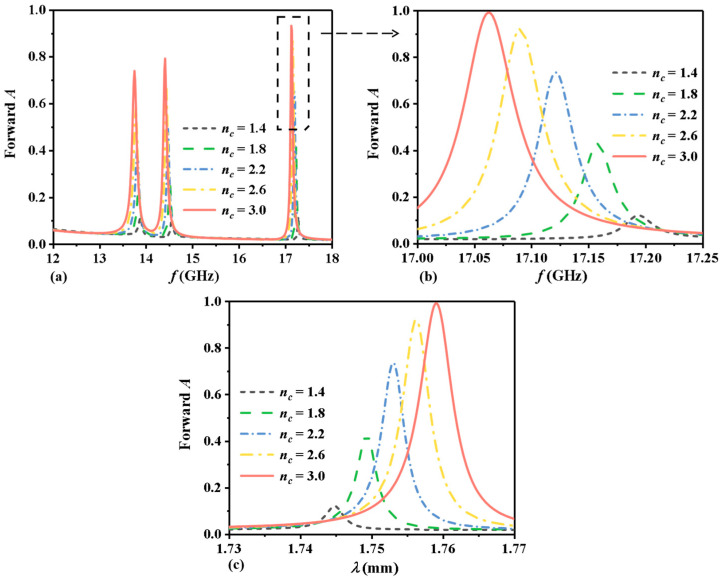
Forward absorption curves corresponding to different RI: (**a**) frequency range from 12 to 18 GHz, (**b**) frequency range from 17.00 to 17.25 GHz, and (**c**) relationship between wavelength and forward absorption curves.

To evaluate the performance of the proposed MPC-based RI sensor, the impact of the different RI of the analyte layer on the forward absorption curves is calculated while keeping other parameters constant (as shown in Table 1). The relevant calculation results are presented in Figure 4. The RI of the analyte layer is denoted by *n_c_*, with a measurement range of 1.2 RIU to 3.8 RIU. To observe the influence of different RI on forward absorption, values of *n_c_* = 1.4 RIU, 1.8 RIU, 2.2 RIU, 2.6 RIU, and 3.0 RIU are selected for analysis. Figure 4a illustrates the variation in the forward absorption curves with changing RI within the range of 12 to 18 GHz. For clearer observation, Figure 4b displays the changes in the value corresponding to the forward absorption peak in our study of 17~17.25 GHz. It is evident that as *n_c_* increases, the peak of forward absorption will redshift, and the absorption rate will gradually increase. Specifically, when *n_c_* = 1.4, the forward absorption peaks at 17.194 GHz with an absorption rate of 0.12. As *n_c_* increases to 1.8, 2.2, and 2.6, the absorption peaks shift to 17.150 GHz, 17.114 GHz, and 17.082 GHz, respectively, with corresponding absorption rates of 0.43, 0.74, and 0.92. At *n_c_* = 3.0, the peak absorption rate reaches 0.99 at 17.054 GHz. Figure 4c illustrates the relationship between wavelength and the absorption curves. As the value of *n_c_* increases, the forward absorption peak shifts toward longer wavelengths, demonstrating a redshift phenomenon. When *n_c_* equals 3.0, the peak absorption rate reaches 0.99 at a wavelength of 1.76 mm.

**Figure 5:**
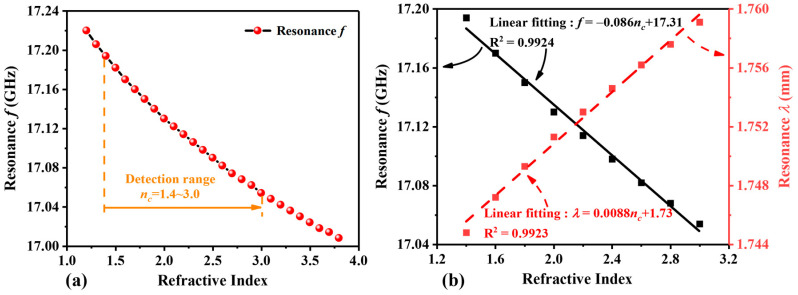
When *N* = 3, (**a**) shows the relationship between the resonance wavelength and RI, and (**b**) shows the linear fitting of the resonance frequency *f* and resonance wavelength *λ* to RI.

Figure 5a illustrates the resonance frequency ranging from 17.220 GHz to 17.008 GHz as the RI of the analyte layer C varies from 1.2 RIU to 3.8 RIU. The results indicate that the resonance frequency of MPC varies with the RI of the analyte layer C and maintains excellent linearity within the range of 1.4~3.0 RIU. Figure 5b depicts the relationship between RI and resonance frequency, showing a negative correlation as the resonance frequency decreases with the increase in *n_c_*. The RI detection range is 1.4~3.0 RIU, with the frequency changing from 17.194 GHz to 17.054 GHz. The linear fitting equation is *f* = −0.086*n_c_* + 17.31, and the S is 86 MHz/RIU. Simultaneously, its linearity *R*^2^ is 0.9924, representing the degree of fit. When *n_c_* = 3.0 RIU, the Q value is 315.8, and the FOM value is 1.66 RIU^−1^. Of course, when considering the relationship between wavelength and RI, the corresponding values are given by the equation *λ* = 0.0088*n_c_* + 1.73, 8.8 µm/RIU and 0.9923.

**Figure 6:**
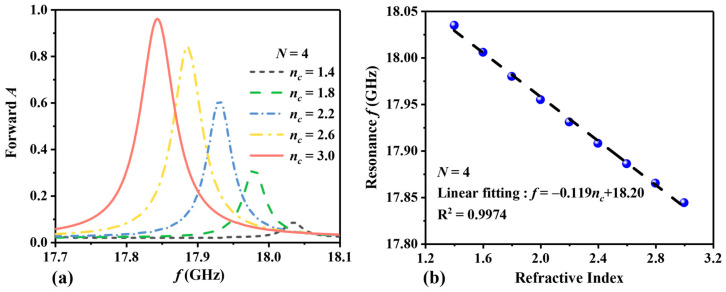
When *N* = 4, (**a**) shows the change in the forward absorption curve with *n_c_*, and (**b**) shows the linear fitting of the resonance frequency with RI. The blue dots represent the sampling points at various RI, while the black dashed lines indicate the fitting curves.

**Figure 7:**
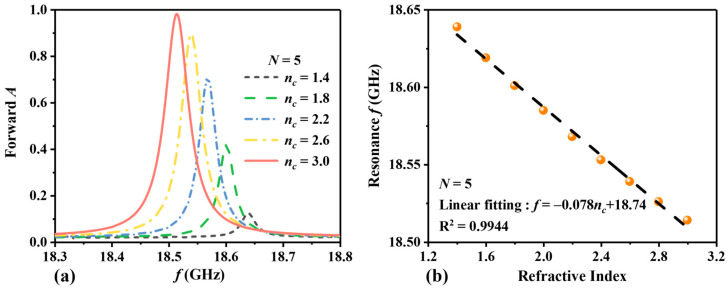
When *N* = 5, (**a**) shows the change in the forward absorption curve with *n_c_*, and (**b**) shows the linear fitting of the resonance frequency with RI. The orange dots represent the sampling points at various RI, while the black dashed lines indicate the fitting curves.

In order to evaluate the effect of the number of periods *N* on the performance of the sensor, we calculate the forward absorption curves for different refractive indices at *N* = 4 and *N* = 5. When *N* = 4, it is shown in Figure 6. Figure 6a illustrates the change in the absorption peak as *n_c_* increases. Overall, it can be observed that as *n_c_* increases, the absorption peak shifts to lower frequencies, and the absorption rate increases. When *n_c_* = 1.4 RIU, the forward absorption peaks at 18.035 GHz with an absorption rate of 0.09. With an increase in *n_c_* to 3.0 RIU, there is a peak absorption rate of 0.96 at 17.844 GHz. In Figure 6b, the resonance frequency and RI are linearly fitted. The detection range of RI remains at 1.4~3.0 RIU, and the resonance frequency shows a negative correlation with RI. The linear fitting equation is *f* = −0.119*n_c_* + 18.20, with an S of 119 MHz/RIU and a linearity *R*^2^ of 0.9974. When *n_c_* = 3.0 RIU, the Q value is 302.4, and the FOM value is 2.02 RIU^−1^. When *N* = 5, the absorption curves are depicted in Figure 7. In Figure 7a, when *n_c_* = 1.4 RIU, the peak of forward absorption occurs at 18.639 GHz, with an absorption rate of 0.12. With an increase in *n_c_* to 3.0 RIU, there is a peak absorption rate of 0.98 at 18.514 GHz. In Figure 7b, RI detection ranges from 1.4 RIU to 3.0 RIU, and the frequency ranges from 18.639 GHz to 18.514 GHz. The linear fitting equation is *f* = −0.078 *n_c_* + 18.74, with an S of 78 MHz/RIU and a linearity *R*^2^ of 0.9944. When *n_c_* = 3.0, the Q value is 363.0, and the FOM value is 1.53 RIU^−1^.

**Figure 8:**
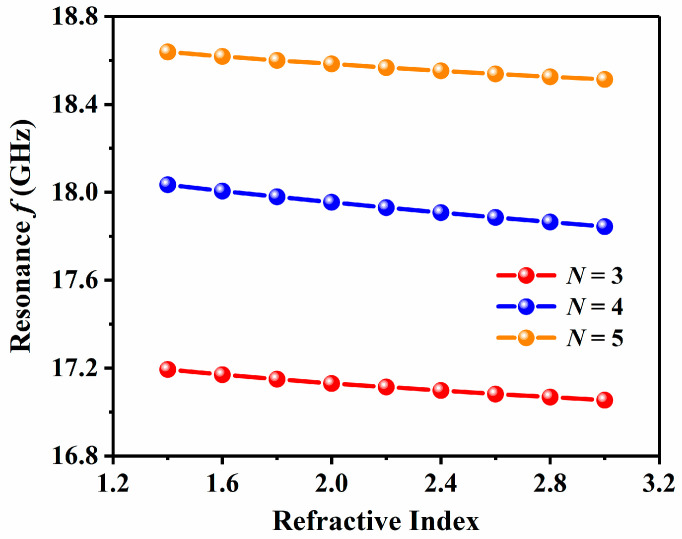
Resonance frequency and period number *N* = 3, *N* = 4, and *N* = 5 are related in various cases.

Under different period numbers *N*, the correlation between resonance frequency and RI is shown in Figure 8, where *N* = 3, 4, and 5 are represented by red, blue, and orange, respectively. It is evident that as *N* increases, the resonance frequency also increases. Based on the analysis, altering the period number *N* will impact the resonance frequency and RI values. Therefore, selecting the appropriate *N* is essential for enhancing sensor performance.

**Figure 9:**
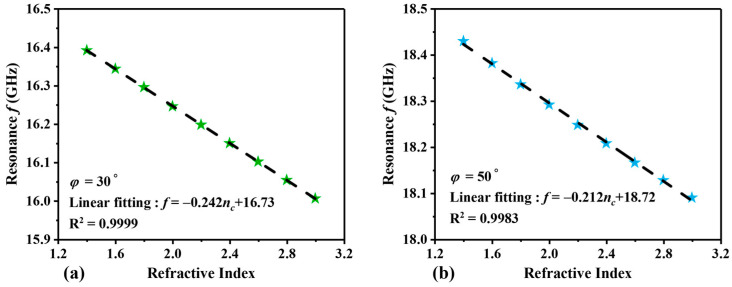
The linear fitting of the resonance frequency with RI at (**a**) *φ* = 30° and (**b**) *φ* = 50°. The green and blue stars represent the sampling points for the different RI, while the black dashed lines indicate the fitted curves.

In addition, we also studied the influence of structural tilt angle *φ* on sensor performance. The relationship between resonance frequency and RI under different tilt angles is shown in Figure 9. In Figure 9a, when *φ* = 30°, the RI detection range is 1.4~3.0 RIU, and the frequency range is 16.006~16.392 GHz. The linear fitting equation is *f* = −0.242*n_c_* + 16.73, the S value is 242 MHz/RIU, and the linearity *R*^2^ is 0.9999. When *n_c_* = 3.0, the Q value is 76.2 and the FOM value is 1.15 RIU^−1^, indicating poor sensor performance. In Figure 9b, with *φ* = 50°, the frequency range is 18.09~18.43 GHz. The linear fitting equation is *f* = −0.212 *n_c_* + 18.72, S is 212 MHz/RIU, and the linearity *R*^2^ is 0.9983. When *n_c_* = 3.0, the Q value is 335 and the FOM value is 3.93 RIU^−1^, showing an improvement in performance compared to *φ* = 30°.

**Figure 10:**
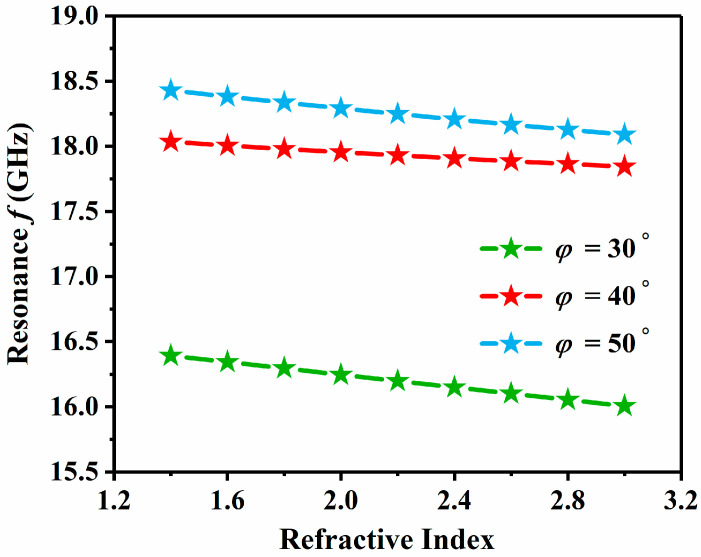
Resonance frequency and tilt angles *φ* = 30°, *φ* = 40°, and *φ* = 50° are related in various cases.

The relationship between resonance frequency and RI at different tilt angles is illustrated in Figure 10, where *φ* = 30°, 40°, and 50° are denoted by green, red, and light blue, respectively. As *φ* increases from 30° to 40°, there is a significant rise in the resonance frequency. At *φ* = 40° and *φ* = 50°, the frequencies are more closely aligned. The sensor’s performance can be manipulated by adjusting the structural tilt angle.

Table 2 presents a comparison with other studies. It is evident that while the MPC-based RI sensor in this paper may not excel in performance, its standout feature lies in its versatility as an asymmetric device, a unique characteristic not observed in prior research findings.

## 4. Conclusions

In summary, this paper presents a novel kind of MPC design that enables the asymmetric propagation of EMWs and wide-range RI detection. The MPC demonstrates unique electromagnetic responses at positive and negative incidences, with a forward absorptivity of 0.94 and a backward transmittance exceeding 0.98 at a resonance frequency of 24.92 GHz. This distinct feature is further evident in multi-frequency AAT after the introduction of the analyte layer, offering detection capabilities. In RI sensing applications, the MPC achieves a broad detection range from 1.4 to 3.0 RIU with a significant absorption peak frequency shift, validating its potential for RI detection. The MPC not only showcases advantages such as versatility, compact size, and a wide measurement range but also indicates promising applications in asymmetric optical components, biosensing, and other advanced technology fields, paving the way for the development of high-performance optical devices.

## Figures and Tables

**Table 1:** Main parameter settings of the model.

Parameter	Symbol	Quantity	
		Ordinary Periodic MPC	MPC Containing the Analyte Layer
Thickness of plasma layer	*d_m_*	0.4 mm	0.6 mm
Thickness of porous silicon layer	*d_f_*	1.0 mm	1.0 mm
Length of hypotenuse	*l*	20.0 mm	20.0 mm
Angle of inclination	*φ*	40°	40°
The incidence angle of EMWs	*θ*	50°	50°
Thickness of analyte layer	*d_c_*	/	1.0 mm

**Table 2:** Comparison with other studies.

Ref.	RI Detection Range	Sensitivity	FOM	Multifunctional Device
[25]	1.0~1.1	2320 nm/RIU	No	No
[29]	1.05~2.05	34.3 GHz/RIU	634	No
[31]	1.000~1.451	25.07 THz/RIU	30.81	No
[40]	1.28~1.44	27,890 nm/RIU	139.78	No
This work	1.4~3.0	86 MHz/RIU	1.66	Yes

## Data Availability

Samples of the compounds are available from the authors.
